# Epigenetic determinants of metastasis

**DOI:** 10.1016/j.molonc.2016.09.008

**Published:** 2017-01-03

**Authors:** Saroor A. Patel, Sakari Vanharanta

**Affiliations:** ^1^ MRC Cancer Unit Hutchison/MRC Research Centre University of Cambridge UK

**Keywords:** cancer, epigenetic, metastasis

## Abstract

Genetic analyses of cancer progression in patient samples and model systems have thus far failed to identify specific mutational drivers of metastasis. Yet, at least in experimental systems, metastatic cancer clones display stable traits that can facilitate progression through the many steps of metastasis. How cancer cells establish and maintain the transcriptional programmes required for metastasis remains mostly unknown. Emerging evidence suggests that metastatic traits may arise from epigenetically altered transcriptional output of the oncogenic signals that drive tumour initiation and early progression. Molecular dissection of such mechanisms remains a central challenge for a comprehensive understanding of the origins of metastasis.

**Abbreviations**
5hmC: 5‐hydroxymethylcytosine
5mC: 5‐methylcytosine
CIMP: CpG island methylator phenotype
CYTIP: cytohesin 1 interacting protein
HDAC: histone deacetylase
HOTAIR: HOX transcript antisense RNA
IDH1: isocitrate dehydrogenase 1
lncRNA: Long non‐coding RNA
MALAT1: metastasis‐associated lung adenocarcinoma transcript‐1
PRC2: polycomb repressive complex 2
RCC: renal cell carcinoma
T‐ALL: T‐cell acute lymphoblastic leukemia
TCAC: tricarboxylic acid cycle

## 1 Introduction

While primary tumours can often be surgically removed, metastases are in most cases incurable. The formation of metastases in distant organs thus represents one of the most devastating aspects of cancer. Large‐scale cancer genome projects have recently catalogued the mutational complements of most human tumours. However, mutations that specifically drive metastatic progression have not been identified. On the other hand, experimental approaches have isolated stable cancer clones that express transcriptional traits associated with metastasis. Many genes have been functionally linked to metastatic progression and several studies have described in detail diverse molecular mechanisms that, in different contexts, endow cancer cells with properties that modulate the rate of metastatic success (Massagué and Obenauf, 2016; Oskarsson *et al*., 2014; Valastyan and Weinberg, 2011). Specific phenotypic traits, dictated by the expression of specific molecules, thus mediate metastatic cancer progression. Little, however, is known about the mechanisms through which cancers acquire metastatic traits. Using specific examples, this review will discuss a model whereby metastatic transcriptional programmes emerge from epigenetic optimization of the oncogenic signals that drive tumour initiation and early progression.

## 2 The metastatic process

Metastases arise through a complex evolutionary process through which cells from a primary tumour eventually form clinically detectable secondary tumours in distant organs. This process is extremely inefficient. The vast majority of cancer cells never leave the primary tumour, and of those that manage to enter the circulation, the vast majority fail to enter distant organs (Baccelli *et al*., 2013; Kienast *et al*., 2010; Luzzi *et al*., 1998). Even of successfully established micro metastases, only a small fraction forms clinically relevant metastatic lesions (Braun *et al*., 2005). Hence, even though often depicted as an orderly sequence of distinct biological steps (Fidler, 2003), metastatic progression is characterized by extreme randomness whereby potentially millions of circulating tumour cells dispatched over several years in the end only form few metastases (Vanharanta and Massagué, 2013). Several distinct barriers for metastatic progression exist. In a simple example, tumour cells originating from epithelia will first have to detach from their native tissue structure, invade the nearby parenchyma, enter the circulation through intravasation, sustain circulatory stress, enter a distal organ through extravasation, survive upon arrival at the distant site, co‐opt the new microenvironment and eventually establish a secondary colony. Additionally, more complicated scenarios can, for example, involve initial invasion and entry into the circulation via the lymphatic system, intermediate metastases in lymph nodes or other sanctuary sites and varying lengths of cellular dormancy of single disseminated cells or micro metastases. Molecular mechanisms that mediate many of these steps have been identified in experimental systems (Massagué and Obenauf, 2016; Oskarsson *et al*., 2014; Valastyan and Weinberg, 2011), and inhibition of a vast array of genes that encode for proteins with various molecular functions can inhibit metastatic progression. The picture emerging from these studies is that metastasis genes do not seem to operate through a shared molecular pathway. Rather, they represent a heterogeneous group of metastasis facilitators that enhance the probability of successful completion of one or more of the metastatic steps via modulating the activity of the oncogenic pathways that also drive the earlier steps of tumorigenesis. How the expression of these traits is regulated remains largely unknown.

## 3 Mutational drivers of metastasis

The inefficiency and distinct biological barriers of metastasis would *a priori* seem to generate an ideal playground for selection to enrich for mutations that confer metastatic capabilities. Indeed, the early genetic models of cancer progression suggested that metastases would be caused by specific mutations (Fearon and Vogelstein, 1990; Nowell, 1976). However, despite extensive efforts, such mutations have not been identified to date (Turajlic and Swanton, 2016; Vogelstein *et al*., 2013). Metastatic cancer clones when compared to the corresponding primary tumour do in most cases contain unique mutations, some of which are predicted to be drivers, but these target genes are already mutated in the primary tumour in other contexts. For example, in pancreatic (Yachida *et al*., 2010), renal (Gerlinger *et al*., 2012) and breast (Yates *et al*., 2015) cancers, distinct clones already present in the primary tumour can be the sources of metastases to different target organs. In prostate cancer (Gundem *et al*., 2015) and melanoma (Sanborn *et al*., 2015), metastatic clones were shown to originate from specific regions of the corresponding primary tumours with sometimes complex relationships between different distant sites, arguing for both parallel and stepwise seeding of metastases as well as continuous seeding of the same distant sites by different cancer clones. On the other hand, in colon cancer, few metastasis‐specific mutations were detected (Jones *et al*., 2008). Finally, a large study comparing metastatic lesions to the corresponding primary tumours from several different cancer types revealed examples of mutational selection during cancer progression, possibly imposed by metastatic bottlenecks and therapies, but no unifying genetic signature of metastasis was identified (Brastianos *et al*., 2015). Some evidence suggests that *TP53* mutations could be important for the emergence of metastatic cancer clones, possibly through effects on genome stability (Turajlic and Swanton, 2016). However, even histologically normal skin can harbour *TP53* mutant clones (Martincorena *et al*., 2015).

Results from experimental metastasis models are in agreement with the data from clinical samples. For example, in genetically engineered mouse models of lung and pancreatic cancer, both parallel and polyclonal seeding of metastases have been observed but the authors did not report enriched mutations in specific genes in the metastatic clones (Maddipati and Stanger, 2015; McFadden *et al*., 2014). Also, human‐derived cancer clones with functionally confirmed metastatic potential can display minimal genetic divergence from the non‐metastatic parental population (Jacob *et al*., 2015), suggesting that the development of metastatic clones from already advanced cancers may not need additional mutations.

From a genetic point of view, no unifying model for metastatic progression thus seems to exist. The reasons for this remain unknown, but several possible explanations have been proposed, including technical issues related to mutation detection and lack of sufficient sample size, the low probability of specific mutations to generate complex phenotypes needed for metastatic competence, and the requirement of already maximal fitness for the development of advanced cancers, after which stochastic metastases would follow without the need for further genetic changes (Frost and Fidler, 1986; Vogelstein *et al*., 2013). It remains possible that further genetic analyses will identify metastasis mutations, at least in some cancers, but accumulating data suggest that such mutations may not be the dominant route for the activation of metastatic traits.

## 4 Epigenetic instability and metastatic progression

If not via genetic alterations, how do cancers acquire metastatic traits? Two general possibilities exist. On the one hand, it may be that no stable traits that drive metastases exist. This view is supported by evidence showing that even metastatic cells can be reprogrammed by embryonic microenvironments, suggesting that at least some of the metastatic traits are reversible and thus most likely not dictated by mutations (Hendrix *et al*., 2007). There is also some evidence for reversal of the aggressive phenotype in lung metastatic foci (Bockhorn *et al*., 2014). On the other hand, at least in experimental systems, stable highly metastatic cancer cell populations with limited genetic divergence from the parental population can be isolated and they express genes that are highly active in patient samples with higher propensity to metastasize, suggesting that pro‐metastatic transcriptional programmes are also present in human tumours (Bos *et al*., 2009; Jacob *et al*., 2015; Minn *et al*., 2005; Vanharanta *et al*., 2013). Many of these genes have been functionally linked to metastatic progression (Massagué and Obenauf, 2016; Oskarsson *et al*., 2014; Valastyan and Weinberg, 2011). This, together with the lack of genetic evidence for metastasis driver mutations, suggests that heritable non‐genetic, i.e. epigenetic, transcriptional programmes underlie metastatic cancer progression.

Several possible molecular mechanisms of epigenetic inheritance have been proposed. The best characterized of these is DNA cytosine methylation, the addition of a methyl group to form 5‐methylcytosine (5mC) at CpG dinucleotides, for which a clear mechanism of inheritance through cell division has been identified (Song *et al*., 2011). Thus, once DNA methylation patterns have been established by a given signal, usually guided by transcription factors with sequence specific DNA‐binding properties (Schübeler, 2015), these marks can be propagated in the absence of the initial stimulus. Conversely, if DNA methylation is lost, either through active or passive demethylation, critical DNA methylation marks can be altered permanently. Modifications of other deoxynucleotides also exist in human cells, but their relevance to epigenetic inheritance remains to be established (Koziol *et al*., 2016). Covalent modifications of the histones represent a second class of molecular alterations that contributes to the maintenance of stable transcriptional programmes (Jenuwein and Allis, 2001). However, unlike DNA methylation, it remains unclear how most of these marks are maintained during replication. Less‐well characterized mechanisms involving non‐coding RNAs also contribute to epigenetic gene regulation (Sabin *et al*., 2013). Finally, stable transcriptional programmes can be supported without any specific heritable changes in DNA methylation or chromatin by autoregulatory transcriptional circuits that after being induced maintain their own transcription (Ptashne, 2007). Identification of such feed‐forward loops as mediators of metastasis is challenging as they can be induced by transient stimuli that may not be present in full‐blown tumours. Yet, they could be critical for the maintenance of cancer phenotypes.

## 5 DNA methylation

DNA methylation is usually associated with transcriptional silencing (Schübeler, 2015). In cancer, widespread hypomethylation is generally observed with only specific regions such as CpG islands being hypermethylated (Berman *et al*., 2012; Hansen *et al*., 2011). Similar observations have been made in many if not most cancer types (Feinberg and Tycko, 2004). Oncogene (e.g. *HRAS*) promoter regions are frequently hypomethylated (Feinberg and Vogelstein, 1983), while tumour suppressor loci such as *TP53*,* APC* and *VHL* are commonly hypermethylated in association with transcriptional silencing (Herman and Baylin, 2003), suggesting that DNA methylation alterations at specific loci can influence tumour progression. The functional relevance of the more widespread DNA hypomethylation in cancer is unknown, but it has been suggested to induce genomic instability (Jones and Gonzalgo, 1997). DNA methylation patterns have also been assessed in metastatic cancer. In prostate cancer, DNA methylation is in general well conserved between primary and corresponding metastatic tumours (Aryee *et al*., 2013), whereas in breast cancer, others have observed metastasis‐specific methylation changes, primarily outside CpG‐rich regions (Reyngold *et al*., 2014). In prostate cancer, there is also considerable intratumoural DNA methylation heterogeneity that correlates well with genomic copy number patterns and metastatic progression (Brocks *et al*., 2014). Thus, there is a significant body of evidence that links aberrations in DNA methylation to tumourigenesis. However, the interpretation of these data is complicated by the observation that the DNA methylation status of promoter CpG islands in a given tumour type tends to reflect the pattern observed in the corresponding tissue of origin (Gebhard *et al*., 2010; Sproul and Meehan, 2013; Sproul *et al*., 2012; Timp and Feinberg, 2013).

**Figure 1:**
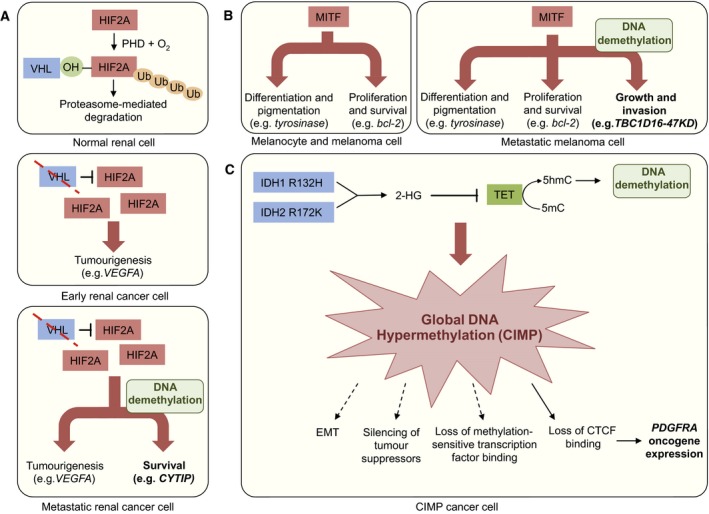
Alterations in DNA methylation patterns as a source of cancer progression traits. (A) Loss of DNA methylation expands the HIF2A tumour‐initiating pathway output in renal cell carcinoma (RCC) to promote metastasis. In the presence of oxygen, HIF2A is normally targeted for proteosomal degradation, but in *VHL* mutant RCC cells HIF2A is constitutively expressed and it drives tumorigenesis. DNA demethylation in metastatic cells increases the HIF2A pathway target gene spectrum to include pro‐metastatic *CYTIP* expression. (B) Melanocyte lineage factor MITF signalling output is expanded through DNA demethylation in support of metastatic progression in melanoma. MITF drives differentiation/pigmentation and proliferation/survival in melanocyte and melanoma cells. Loss of DNA methylation allows MITF to bind additional gene promoters and induce expression of metastasis‐promoting *TBC1D16*. (C) Metabolic alterations can induce global alterations in DNA methylation. In glioblastoma, IDH1/2 mutants produce the oncometabolite 2‐hydroxyglutarate (2‐HG) which inhibits the activity of TET enzymes. TETs normally mediate demethylation of DNA by converting 5‐methylcytocine (5mC) to 5‐hydroxymethylcytosine (5hmC). Thus, the accumulation of 2‐HG leads to increased DNA methylation resulting in the global CpG island hypermethylator phenotype (CIMP). This can lead to tumour suppressor silencing or loss of the binding of methylation sensitive DNA‐binding factors such as CTCF. CTCF functions as an insulator protein that demarcates chromatin domains. In glioblastoma, CIMP‐induced loss of CTCF binding can allow aberrant *PDFGRA* activation. Thus, unspecific large‐scale alterations in DNA methylation can result in specific cancer phenotypes. Similar mechanism could activate metastasis genes as well.

Several examples link aberrations in DNA methylation to the acquisition of functionally important metastatic traits (Fig. 1). In renal cell carcinoma (RCC), increased global DNA methylation is associated with poor patient outcome (The Cancer Genome Atlas Network, 2013). However, experimental analysis has identified functional mediators of RCC metastasis that are activated via DNA demethylation events. For example, cytohesin 1 interacting protein (CYTIP) and S100 calcium binding protein A4 are highly expressed and demethylated in metastatic RCC subpopulations and they functionally mediate metastatic colonization (Lopez‐Lago *et al*., 2010; Vanharanta *et al*., 2013). Additionally, hypermethylation of the *CYTIP* locus is associated with good patient survival (Gevaert *et al*., 2015). Without an active transcriptional programme, loss of DNA methylation does not lead to gene activation. Indeed, *CYTIP* expression is driven by the RCC‐initiating VHL‐HIF pathway (Fig. 1A) (Vanharanta *et al*., 2013). A similar mechanism has been implicated in melanoma progression, whereby loss of DNA methylation facilitates the expression of a variant transcript of the Rab GTPase‐activating protein TBC1D16 in support of metastasis (Vizoso *et al*., 2015). Once released by repression of DNA methylation, *TBC1D16* expression is driven by the core melanocyte lineage factor MITF (Fig. 1B). DNA hypomethylation has also been linked to osteosarcoma metastasis through the induction of *IRX1*, however the pathway that drives gene expression in this case remains unidentified (Lu *et al*., 2015). In these examples, the mechanism through which DNA methylation is lost remains unknown. It could be through passive stochastic dilution of methylated CpGs during cell division, but accumulating evidence suggests that active DNA demethylation can also be involved in the acquisition of invasive and metastatic phenotypes (Munoz *et al*., 2013).

DNA methylation can also silence metastasis suppressor genes. Global profiling of matched cell lines from metastasis and primary tumour identified CDH11 as a target of silencing via DNA methylation and subsequent functional analysis supported a role for CDH11 in metastasis suppression (Carmona *et al*., 2012). Similarly, several microRNAs have been shown to undergo methylation‐dependent silencing in metastatic cancer (Lujambio *et al*., 2008). The genomic locus for the metastasis suppressor miR‐335 is epigenetically silenced and subsequently also deleted in metastatic breast cancer; however, this is through maternal imprinting (Png *et al*., 2011). Epigenetic silencing of miR‐335 is thus not cancer‐associated, but rather, the silencing of a gene during development serves essentially as the first inactivating hit of a tumour suppressor.

A CpG island methylator phenotype (CIMP) has been described in many cancer types (Issa, 2004). Analysis of human data sets have demonstrated that in some cancers, such as prostate cancer, CIMP is associated with poor outcome (Gu *et al*., 2015), whereas in others, such as breast cancer and glioblastoma, CIMP seems to correlate with increased patient survival (Fang *et al*., 2011; Turcan *et al*., 2012), possibly because CIMP‐induced stable gene repression may reduce the probability of the acquisition of aggressive cancer phenotypes (Sproul and Meehan, 2013). In glioblastoma, CIMP has been linked to the metabolic alterations caused by mutations in the tricarboxylic acid cycle (TCAC) enzyme isocitrate dehydrogenase (IDH1) (Turcan *et al*., 2012). In this model, increased levels of the oncometabolite 2‐hydroxyglutarate inhibit the activity of the TET enzymes that are important for the conversion of 5mC to 5‐hydroxymethylcytosine (5hmC), an intermediate step for active demethylation, thus leading to global increase in DNA methylation (He *et al*., 2011; Xu *et al*., 2011) (Fig. 1C). Interestingly, DNA methylation in this context can change the affinity of the chromatin insulator protein CTCF to specific target loci, leading to altered chromatin domain structure and activation of oncogenic drivers such as PDGFRA (Flavahan *et al*., 2016). The extent to which similar CTCF repositioning is involved in other cancer contexts, including metastasis, remains unclear, but there is evidence that CTCF/cohesin binding sites can accumulate mutations in several cancers (Katainen *et al*., 2015). In addition to IDH1, loss of other TCAC enzymes such as FH and SDH can also induce aberrant DNA methylation patterns (Letouze *et al*., 2013; Sciacovelli *et al*., 2016), and microenvironmental factors such as hypoxia can lead to global DNA hypermethylation with possible cancer promoting consequences (Thienpont *et al*., 2016). As shown in breast cancer, microRNA‐mediated silencing of TET enzymes can similarly lead to changes in DNA methylation and consequent activation of metastasis genes (Song *et al*., 2013). In breast cancer, widespread DNA methylation that promotes metastasis may also be induced by receptor tyrosine kinase activation and consequent induction of the methyl‐CpG binding domain protein 4 (Cunha *et al*., 2014). On the other hand, in prostate cancer, CIMP has been linked to an increased activity of the chromatin reader BAZ2A that interacts with the polycomb repressive complex 2 (PRC2) (Gu *et al*., 2015). Finally, in colorectal cancer oncogenic KRAS^G13D^ seems to maintain CIMP and consequent suppression of tumour suppressors through the expression of sequence specific DNA‐binding proteins and associated modulators of gene repression (Serra *et al*., 2014), demonstrating that specific signalling pathways can also induce global changes in DNA methylation.

It is unclear what mechanisms target DNA methylation or demethylation events to specific loci during metastatic cancer progression. The activation of oncogenic pathways can target DNA methyltransferases to tumour suppressor loci and at least in some cases, active signalling is required for the maintenance of gene silencing and DNA methylation (Gazin *et al*., 2007; Serra *et al*., 2014). It could be that DNA methylation changes in metastasis also reflect alterations in the activities of transcription factors that have DNA‐binding specificities. Indeed, experimental evidence suggests that DNA sequences play an instructive role in determining DNA methylation patterns in normal cells (Krebs *et al*., 2014). However, the role of local sequence characteristics in determining the DNA methylation status in cancer is less clear (Krebs *et al*., 2014) and specific pro‐metastatic changes could also emerge from the stochastic alterations in global DNA methylation patterns through selection (Hansen *et al*., 2011; Landan *et al*., 2012; Timp and Feinberg, 2013).

## 6 Chromatin alterations

While there is evidence that transcription factors determine the genomic distribution of tissue specific histone marks (Benveniste *et al*., 2014), at least some repressive histone modifications seem to be heritable through the cell cycle (Hansen *et al*., 2008; Hathaway *et al*., 2012; Margueron *et al*., 2009). Stable chromatin alterations could therefore be another way for cancer cells to lock in metastatic phenotypes. The frequent mutations in various chromatin factors and histones in cancer have indisputably linked chromatin biology to tumorigenesis (Shen and Laird, 2013). Mutations in some of these genes, such as *PBRM1* and *BAP1* in RCC, are associated with poor patient outcome (Joseph *et al*., 2016; Pawlowski *et al*., 2013), indicating that their downstream pathways may also contribute to cancer progression and metastasis.

### 6.1 The polycomb repressive complex 2

**Figure 2:**
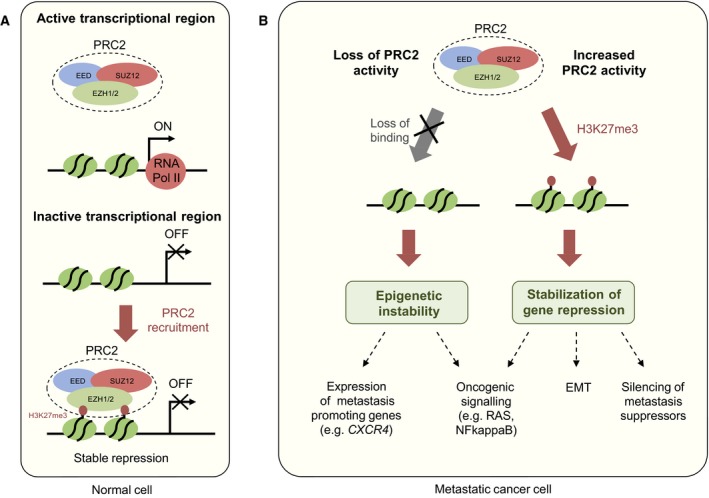
Aberrant polycomb repressive complex 2 activity as a source of metastatic cancer phenotypes. (A) In normal cells, PRC2 accumulates at inactive promoters and it functions as a stabilizer of gene silencing through the deposition of the H3K27me3 repressive mark. (B) In tumour cells, depending on the context, PRC2 can both promote and inhibit cancer progression. In some cancers, PRC2 is important for the stable suppression of genes that inhibit metastasis. On the other hand, reduced PRC2 activity can in some contexts promote metastasis by inducing epigenetic instability, which leads to increased transcriptional plasticity. This can facilitate the activation of pro‐metastatic genes such as CXCR4.

The PRC2 is a critical regulator of gene repression in multiple biological contexts (Margueron and Reinberg, 2011). It consists of three core subunits, EED, SUZ12, the catalytic subunit EZH1/EZH2, and several associated factors. EZH1/EZH2 can deposit trimethylation marks on histone H3 lysine 27 (H3K27me3), which can be recognized by the chromatin reader subunit EED (Margueron *et al*., 2009). This is thought to allow the mark to be propagated through cell division. There is evidence that deposition of PRC2 at given genomic loci is passive: inactive promoters naturally acquire PRC2 and H3K27me3, which then stabilizes the inactive state (Jermann *et al*., 2014; Riising *et al*., 2014) (Fig. 2A). Interestingly, both activating and inactivating mutations in EZH2 have been identified in different cancers (Cerami *et al*., 2012; Ernst *et al*., 2010; McCabe *et al*., 2012; Souroullas *et al*., 2016). Moreover, EZH2 is often upregulated in various cancers and the expression of EZH2 often correlates with poor patient outcome (Chase and Cross, 2011; Chen *et al*., 2015); although this could, at least in some cases, be simply reflecting the higher proliferative phenotype associated with cancer progression (Wassef *et al*., 2015). However, EZH2 activity is critical for EMT in breast cancer (Malouf *et al*., 2013; Tiwari *et al*., 2013) and metastatic progression of melanoma (Zingg *et al*., 2015). Similarly, EZH2‐mediated silencing of a Ras GTPase‐activating protein enhances RAS and NF‐kappaB signalling in metastatic prostate cancer (Min *et al*., 2010). On the other hand, loss of PRC2 activity has also been linked to metastatic progression. For example, expression of the chemokine receptor *CXCR4* is suppressed by PRC2 in RCC, and loss of this suppression facilitates *CXCR4* expression and increases the metastatic fitness of RCC cells (Vanharanta *et al*., 2013). In this case, *CXCR4* expression is also dependent on the activity of the VHL‐HIF pathway that drives RCC induction and early progression, yet HIF2A activity alone is not sufficient for *CXCR4* expression. What leads to PRC2 eviction from the *CXCR4* locus during RCC progression remains unknown, but it is noteworthy that low global levels of H3K27me3 seem to be associated with poor patient outcome in RCC (Rogenhofer *et al*., 2012). PRC2 loss can also potentiate oncogenic RAS signalling in specific genetic contexts, leading to potential therapeutic vulnerabilities (De Raedt *et al*., 2014). Interestingly, even transient reduction of PRC2 activity can induce stable expression of its target genes, suggesting that PRC2 loss can promote epigenetic instability that may contribute to cancer progression (Wassef *et al*., 2015). Analogously, PRC2 inhibition can support the emergence of drug resistant cancer clones (Rathert *et al*., 2015). Thus, while PRC2 function in some cancers seems to be critical for the suppression of genes that inhibit metastasis, there is also strong evidence that loss of PRC2 promotes metastasis in other contexts, possibly by increasing transcriptional plasticity (Fig. 2B).

### 6.2 The SWI/SNF complex

SWI/SNF is a multi‐subunit ATP‐dependent chromatin remodelling complex that plays a critical role in several developmental processes (Wilson and Roberts, 2011). In humans, there are 29 genes that encode the 15 SWI/SNF subunits in a partially tissue specific manner (Kadoch and Crabtree, 2015). Strikingly, > 20% of cancers carry mutations in SWI/SNF complex genes, making it the most frequently mutated chromatin regulatory pathway in human cancer (Kadoch *et al*., 2013). The mutations show strong tissue specificity, indicating that different SWI/SNF variants modulate the transcriptional output of tissue specific oncogenic and tumour suppressive pathways. Most SWI/SNF mutations seem to be inactivating (Kadoch and Crabtree, 2015). Interestingly, mutations in SWI/SNF complex members may result in specific molecular vulnerabilities that could be exploited therapeutically (Bitler *et al*., 2015; Helming *et al*., 2014). While the genetic evidence linking SWI/SNF dysregulation to carcinogenesis is strong, fairly little is known about the mechanisms that link SWI/SNF to tumorigenesis. Similarly, it remains unclear how SWI/SNF alterations contribute to metastasis. In RCC, data from multi‐region tumour sequencing suggest that mutations in the SWI/SNF member PBRM1 occur early during tumorigenesis (Gerlinger *et al*., 2014). Yet, *PBRM1* mutations seem to be associated with poor patient outcome in these tumours (Pawlowski *et al*., 2013), suggesting that PBRM1 may modulate the probability of RCC cells acquiring metastatic traits at a later stage of tumour progression. Conversely, some evidence suggests that in breast cancer SWI/SNF activity may promote metastatic progression (Wang *et al*., 2014).

### 6.3 Other chromatin factors

In addition to the Polycomb and SWI/SNF complexes, multiple other chromatin regulators have been linked to metastatic progression. For example, the inhibitory nuclear corepressor 1, a member of histone deacetylase (HDAC) complexes that is known to suppress nuclear receptor activity, suppresses metastatic hepatocellular carcinoma progression by inhibiting the expression of multiple metastasis genes (Martinez‐Iglesias *et al*., 2016). Similarly, the related members of another HDAC complex, BRMS1 and BRMS1L, inhibit breast cancer metastasis by suppressing multiple transcriptional targets (Gong *et al*., 2014; Hurst, 2012). The histone H3 lysine 4 demethylase LSD1, a member of the multifunctional nucleosome remodelling and deacetylase complex, can also inhibit metastasis (Wang *et al*., 2009). A more specific metastasis suppressive role has been described for the (NAD)+‐dependent HDAC SIRT6 in pancreatic ductal adenocarcinoma (Kugel *et al*., 2016); the effects of SIRT6 loss seem to be largely mediated through induction of the RNA‐binding protein Lin28b. Tumours with low SIRT6 expression are highly dependent on Lin28b, highlighting the interesting possibility that changes in the activity of specific HDACs could lead to cancer cell dependencies with therapeutic potential even in the metastatic setting. On the other hand, other HDACs, such as SIRT1, have positive effects on metastatic progression in prostate cancer (Byles *et al*., 2012). While these examples clearly demonstrate the relevance of HDAC complexes for metastatic cancer progression, they also highlight the context specific nature of HDAC phenotypes.

In addition to LSD1, several other histone demethylases and methyltransferases have been implicated in metastatic cancer progression. Activation of the histone H3 lysine 9 demethylase PHF2 can lead to mesenchymal‐to‐epithelial transition, i.e. the reversal of EMT, thus leading to reduced tumour‐initiating capacity in breast cancer (Pattabiraman *et al*., 2016). Histone H3 lysine 4 demethylase KDM5A is critical for breast cancer progression and metastasis by facilitating the expression *TNC* and other genes, but the mechanism is independent of the demethylase activity of KDM5A (Cao *et al*., 2014). KDM2A demethylates H3K36me2 in lung cancer, which reduces the expression of several genes such as *HDAC3* and *DUSP3* leading to increased invasiveness (Dhar *et al*., 2014; Wagner *et al*., 2013). JMJD2C/KDM4C interacts with HIF1A to enhance target gene activation and promote breast cancer progression and metastasis by demethylating H3 lysine 9 near several HIF1A target genes (Luo *et al*., 2012). The H3K79 methyltransferase DOT1L promotes breast cancer metastasis by activating many EMT mediators in collaboration with c‐MYC (Cho *et al*., 2015). The H3K4 methyltransferases KMT2A and KMT2B, both members of the MLL complex, collaborate with the androgen receptor to promote prostate cancer progression (Malik *et al*., 2015). Finally, altered expression of histones has been linked to metastatic progression; the histone variant macroH2A inhibits melanoma progression through suppression of CDK8 expression (Kapoor *et al*., 2010).

Collectively these selected examples highlight the fact that alterations in the activity of various different chromatin regulators that lack DNA‐binding specificities are functionally linked to metastatic progression. While for some of them a transcriptional programme that confers specificity has been identified, in many cases it is unclear why a particular chromatin regulator is important in a given cancer context. However, the emerging picture suggests that chromatin factor specificity in different metastatic phenotypes is likely to reflect the underlying oncogenic driver pathways. As many chromatin factors are potentially druggable, it is of significant interest to identify the chromatin regulatory dependencies of the core oncogenic programmes that drive metastasis in different cancers.

## 7 Long non‐coding RNAs

**Figure 3:**
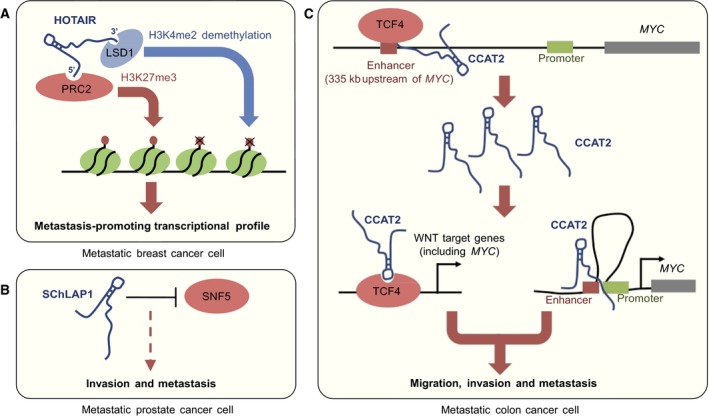
Long non‐coding RNAs in the epigenetic regulation of metastasis. (A) LncRNA HOTAIR found in breast cancer metastasis binds and activates PRC2 and LSD1 to remodel the histone methylation landscape. This can induce a pro‐metastatic gene expression profile. (B) SChLAP1 lncRNA expression is found in metastatic prostate cancer cells. Mechanistically, SChLAP1 binds and antagonizes SNF5, a component of the SWI/SNF complex. (C) CCAT2 is transcribed from the enhancer region 335 kb upstream of *MYC*. Accumulation of CCAT2 in metastatic colon cancer cells stimulates expression of WNT target genes including *MYC* by promoting TCF4 binding. CCAT2 also mediates enhancer‐promoter interaction upstream of *MYC* to increase *MYC* expression in metastatic cells.

Long non‐coding RNAs (lncRNAs) are emerging as important regulators of various biological processes, including chromatin remodelling and DNA methylation (Sabin *et al*., 2013). Increasing evidence is also supporting their role in the control of metastatic progression. For example, HOX transcript antisense RNA (HOTAIR) is strongly upregulated in breast tumours, and it is a powerful predictor of subsequent metastasis and death (Gupta *et al*., 2010). HOTAIR binds directly to PRC2 and LSD1 inducing their genome‐wide retargeting and an altered gene expression profile in cancer cells (Gupta *et al*., 2010; Tsai *et al*., 2010) (Fig. 3A). In prostate cancer, high expression of the lncRNA SChLAP1 correlates with aggressive tumour phenotypes and metastasis, and experimental evidence suggests that SChLAP1 antagonizes the tumour suppressive functions of the SWI/SNF complex (Prensner *et al*., 2013) (Fig. 3B). In metastatic non‐small cell lung cancer, increased expression of metastasis‐associated lung adenocarcinoma transcript‐1 (MALAT1) correlates with poor prognosis (Ji *et al*., 2003) and inhibition of MALAT1 expression reduces lung cancer metastasis (Gutschner *et al*., 2013). Additionally, lncRNA BCAR4 controls the epigenetic regulation of Hedgehog/GLI2 transcriptional activation in support of breast cancer progression (Xing *et al*., 2014), whereas CCAT2 modulates WNT and MYC activity in colon cancer leading to increased metastasis (Ling *et al*., 2013) (Fig. 3C). Non‐coding enhancer RNAs (eRNAs) are also emerging as important regulators of oncogenic signalling (Li *et al*., 2013), but their role in metastasis remains unexplored. Current evidence thus has implicated several lncRNAs in metastasis, and evidence from other contexts suggests that lncRNAs can influence the epigenetic machinery. Also, through sequence complementarity with DNA or nascent RNA, lncRNAs could in principle direct epigenetic regulators to specific genomic loci. If and how such mechanism affect the epigenetic inheritance of stable metastatic traits is an interesting avenue for future exploration.

## 8 Autoregulatory transcriptional circuits

As demonstrated by cellular reprogramming experiments, self‐sustaining transcriptional networks define cell identities (Hochedlinger and Jaenisch, 2015). Different tissues, including cancers, are thus characterized by unique gene expression profiles and chromatin landscapes (Hnisz *et al*., 2013). Accordingly, sporadic cancers arising in different tissues harbour distinct patterns of mutations (Vogelstein *et al*., 2013), and germline mutations in hereditary cancer syndromes predispose to specific tumour types (Vogelstein and Kinzler, 1998). Hence, while mutations can dictate cancer phenotypes, gene regulatory patterns can determine whether or not specific mutations confer selective advantage. In general, little is known about the transcriptional programmes that define cell states in most cancer types. However, several predictions have been made (Saint‐Andre *et al*., 2016) and some have been functionally characterized. In glioblastoma, functional dissection of tumour initiation potential revealed a transcriptional network consisting of four transcription factors, OLIG2, POU3F2, SALL2 and SOX2, as critical enforcers of stem cell identity (Suva *et al*., 2014). These factors were expressed in a substantial proportion of glioblastoma cells from patient samples, suggesting that the core transcriptional network of glioblastomas was not a feature of rare cancer stem cells (Patel *et al*., 2014). In T‐cell acute lymphoblastic leukemia (T‐ALL), specific non‐coding mutations upstream of the *TAL1* oncogene are capable of generating a self‐sustaining transcriptional loop, which involves several T‐ALL transcription factors such as MYB, GATA3, RUNX1 and TAL1 itself (Mansour *et al*., 2014). Interestingly, cancer‐defining transcriptional programmes can be extremely sensitive to perturbations in the activity of specific transcription factors or chromatin binding proteins (Loven *et al*., 2013). For example, in acute myeloid leukaemia, both enhanced and reduced activity of critical transcriptional regulators, such as IRF8, CEBPA, ETV6 and FOSL2, and the inhibition of the broadly targeted chromatin reader BRD4 reduce cellular fitness (Pelish *et al*., 2015; Zuber *et al*., 2011). Several transcription factors have also been linked to either increased or decreased metastatic propensity (Brady *et al*., 2016; Cheung *et al*., 2013; Denny *et al*., 2016; Winslow *et al*., 2011). It is possible that these or other factors contribute to transcriptional circuits that could autonomously maintain metastatic transcriptional programmes. Some studies have indeed explored this possibility (Lee *et al*., 2014), but the extent and nature of such mechanisms as regulators of metastatic progression remain mostly unknown.

## 9 Oncogenic pathways as drivers of metastasis

**Figure 4:**
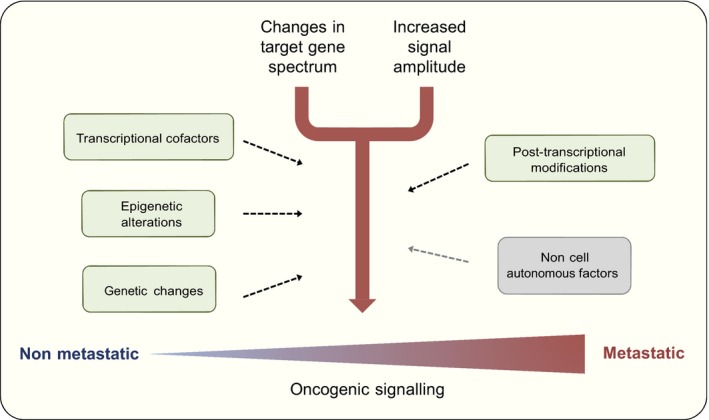
Optimization of oncogenic signal output facilitates metastatic cancer progression. Thus far, no metastasis‐specific genetic pathways have been identified. This suggests a model whereby metastatic progression is supported by the same oncogenic pathways that drive tumour initiation and early progression. However, the output of these pathways is not constant but evolves during cancer progression in support of the most aggressive tumour phenotypes. In a mutually non‐exclusive manner, this modulation of pathway output can be both quantitative and qualitative. For example, during metastatic progression a given pathway may become more active generally through increased expression of its core effectors, but there may also be more specific alterations in the expression of individual target genes. At the molecular level, this fine‐tuning of oncogenic signalling can occur through multiple mechanism including genetic and epigenetic alterations, changes in the abundance of transcriptional cofactors and post‐transcriptional modulation of gene products. Non‐cell autonomous factors, whereby signalling is regulated through interaction with stromal components and extracellular matrix, can also modulate the strength of oncogenic signalling in cancer cells and consequently increase metastatic fitness.

As can be seen from the examples above, epigenetic alterations are intimately linked to metastasis. However, there is no general pattern that would universally connect a specific epigenetic modality to metastatic progression; each regulatory mechanism can either promote or inhibit metastasis depending on the context. This is logical, as the epigenetic regulatory mechanisms are general modifiers of gene expression and their target genes are dependent on the transcriptional state of the cell. Thus, the epigenetic modifications that promote metastatic progression do so by modifying the output of the already activated transcriptional programmes (Fig. 4). The drivers of these programmes are either activated specifically in the cancer cells through oncogenic mutations, or they represent endogenous linage and other factors that are induced without cancer‐specific oncogenic alterations. Examples of both have been described. Tumour‐initiating mutations in *VHL* lead to the activation of the transcription factor HIF2A, but this does not automatically lead to the acquisition of a metastatic phenotype. However, epigenetic modulation of the HIF2A transcriptional output can later on lead to the expression of pro‐metastatic genes (Vanharanta *et al*., 2013). On the other hand, the hormone‐dependent transcription factor networks that are critical for the normal development and homoeostasis of the breast and prostate epithelium, respectively, drive the expression of pro‐metastatic genes in advanced cancers arising in these tissues (Goodwin *et al*., 2015; Ross‐Innes *et al*., 2012). Similarly, the transcriptional output of the linage factor MITF is epigenetically modulated in support of melanoma progression (Vizoso *et al*., 2015). Most epigenetic alterations associated with metastatic progression seem to operate in an analogous manner, but it is in general unknown what causes these changes in the epigenetic landscape. In a mouse model of small cell lung cancer, the activation of a single transcription factor can induce widespread alterations in chromatin accessibility in association with metastatic progression (Denny *et al*., 2016). Similar mechanisms could activate metastasis genes in other cancers as well.

The general concept of qualitatively and quantitatively altered oncogenic signalling output as a driver of metastatic progression is supported by multiple levels of evidence beyond epigenetic transcriptional modulation. First, the lack of mutations specifically associated with metastatic progression indicates that the activation of general oncogenic pathways usually takes place at a pre‐metastatic stage of tumour development (Vogelstein *et al*., 2013). Second, several micro‐RNAs and other mRNA modulatory factors have been functionally linked to metastatic progression (Fish *et al*., 2016; Goodarzi *et al*., 2014; Pencheva and Tavazoie, 2013; Vanharanta *et al*., 2014). Rather than activating specific pathways, post‐transcriptional regulation of mRNA abundance would also fine‐tune the transcriptional output of already activated cellular signalling networks. Finally, several non‐cell autonomous mechanisms of metastatic cancer progression described in multiple model systems lead to hyperactivation of one or more of the core oncogenic pathways in the metastatic cell (Oskarsson *et al*., 2014). For example, interactions between breast cancer cells and stromal components can amplify survival signals downstream of Akt in several metastatic contexts (Chen *et al*., 2011; Zhang *et al*., 2009), but the pathway is often activated already in primary breast tumours (The Cancer Genome Atlas Network, 2012).

## 10 Future perspective

Instead of through the activation of specific metastasis pathways, the metastatic phenotype seems to be acquired through complex fine‐tuning of the amplitude and target gene spectra of the core pathways that drive tumour initiation and early progression. Some of these pathways are activated by mutations, others by non‐mutated lineage factors. In this context, epigenetic alterations do not drive cancer progression but rather facilitate the establishment of the most aggressive phenotypes via selection. The metastatic potential is a continuum and it is acquired by multiple independent alterations in a process akin to genetic evolutionary tinkering (Jacob, 1977). However, even though the process may be random, this epigenomic tinkering tends to follow similar evolutionary paths in different tumours of the same tissue of origin, as is evidenced by the shared transcriptional patterns associated with metastatic progression in different patients and experimental systems. In this model, the intrinsic metastatic fitness of a given cell is dictated by the combined net effect of all the active pathways in that cell. It may therefore be difficult to precisely determine the stage of tumour progression at which the metastatic phenotype is acquired: some metastasis genes may be activated early and others late. Despite this concept of metastatic progression being supported by multiple lines of clinical and experimental evidence, several open questions remain:

- In addition to changes in DNA methylation, which clearly can allow a transcriptional regulator to access new target loci, the general mechanisms through which active oncogenic programmes are modulated during metastatic progression remain unknown. For example, can alterations in higher order chromatin structure promote metastasis? What is the role of transcriptional enhancers in metastatic progression?
- Some chromatin regulators, such as the PRC2 complex, act as buffers that stabilize transcriptional programmes and consequently cell fates. Reduced PRC2 function could thus promote metastatic progression indirectly by increasing transcriptional variability from which aggressive clones could emerge. Transcriptional variability has been linked to higher likelihood of metastatic relapse (Nguyen *et al*., 2016). However, the origins of transcriptional noise that support metastatic progression remain poorly understood.
- The tumour microenvironment is an integral player in cancer progression and several examples of pro‐metastatic cancer‐stroma signalling loops have been identified. Stromal signals can also direct selection towards metastasis (Zhang *et al*., 2013). Whether or not transient microenvironmental cues are able to stably reprogramme cancer cell transcriptomes remains unclear, however.
- Mutations in several enzymes can change the metabolic milieu in cancer cells. This has been linked to widespread alterations in DNA methylation, as exemplified by IDH1 mutations in glioblastoma (Turcan *et al*., 2012). The contribution of other metabolic alterations to epigenetic control of cancer progression remains largely unexplored. Could therapeutic perturbation of metabolic pathways modulate cancer cell transcriptomes and consequently affect the probability of metastatic progression?
- Developmental genetic programmes are remarkably robust, faithfully recapitulating complex phenotypes. Cancers, on the other hand, seem to benefit from genetic and epigenetic instability. It would be important to know, what kind of consequences does epigenetic instability have on cancer cell fitness. Does it only increase plasticity or could it also lead to vulnerabilities that could be exploited therapeutically?
